# The effect of conditioning regimen and prescribed medications on hyposalivation in haematopoietic cell transplantation (HCT) patients: an 18-month prospective longitudinal study

**DOI:** 10.1007/s00784-023-05327-1

**Published:** 2023-10-18

**Authors:** Marjolein S. Bulthuis, Lucky L. A. van Gennip, Renske Z. Thomas, Ewald M. Bronkhorst, Alexa M. G. A. Laheij, Judith E. Raber-Durlacher, Frederik R. Rozema, Michael T. Brennan, Inger von Bültzingslöwen, Nicole M. A. Blijlevens, Marie-Charlotte D. N. J. M. Huysmans, Stephanie J. M. van Leeuwen

**Affiliations:** 1 Department of Dentistry, Radboud University Medical Center, Nijmegen, The Netherlands; 2 Department of Oral Medicine, Academic Centre for Dentistry Amsterdam, University of Amsterdam and VU University, Amsterdam, The Netherlands; 3 Department of Preventive Dentistry, Academic Centre for Dentistry Amsterdam, University of Amsterdam and VU University, Amsterdam, The Netherlands; 4 Department of Oral and Maxillofacial Surgery, Amsterdam UMC, University of Amsterdam, Amsterdam, The Netherlands; 5 Department of Oral Medicine/Oral & Maxillofacial Surgery, Atrium Health Carolinas Medical Center, Charlotte, NC; Department of Otolaryngology/Head & Neck Surgery, Wake Forest University School of Medicine, Winston-Salem, NC USA; 6 Department of Oral Microbiology and Immunology, Institute of Odontology, The Sahlgrenska Academy, University of Gothenburg, Gothenburg, Sweden; 7 Department of Hematology, Radboud University Medical Center, Nijmegen, The Netherlands

**Keywords:** Haematopoietic cell transplantation, Hyposalivation, Salivary flow rate, Medications

## Abstract

**Objectives:**

Haematopoietic cell transplantation (HCT) preceded by a conditioning regimen is an established treatment option for (non)malignant haematologic disorders. We aim to describe the development of hyposalivation over time in HCT recipients, and determine risk indicators.

**Materials and methods:**

A multi-centre prospective longitudinal observational study was conducted. Unstimulated (UWS) and stimulated (SWS) whole saliva was collected before HCT, early post-HCT, and after 3, 6, 12, and 18 months. The effect of type of transplantation (allogeneic vs autologous) and intensity (full vs reduced) of the conditioning regimen on hyposalivation (UWS < 0.2 mL/min; SWS < 0.7 mL/min) was explored.

**Results:**

A total of 125 HCT recipients were included. More than half of the patients had hyposalivation early post-HCT; a quarter still had hyposalivation after 12 months. The conditioning intensity was a risk indicator in the development of hyposalivation of both UWS (OR: 3.9, 95% CI: 1.6–10.6) and SWS (OR: 8.2, 95% CI: 2.9–24.6). After 3 and 12 months, this effect was not statistically significant anymore.

**Conclusions:**

Hyposalivation affects the majority of patients early post-HCT. The conditioning intensity and the type of transplantation were significant risk indicators in the development of hyposalivation. The number of prescribed medications, total body irradiation as part of the conditioning regimen and oral mucosal graft-versus-host disease did not influence hyposalivation significantly.

**Clinical relevance:**

Because of the high prevalence of hyposalivation, HCT recipients will have an increased risk of oral complications. It might be reasonable to plan additional check-ups in the dental practice and consider additional preventive strategies.

## Introduction

Haematopoietic cell transplantation (HCT) is a potentially curative treatment for haematologic cancers and many other non-malignant disorders [1]. In HCT, stem cells are either harvested from the patient (autologous HCT) or from a donor (allogeneic HCT). The stem cell infusion is preceded by a conditioning regimen, consisting of chemotherapy with or without total body irradiation (TBI). During the past decades, clinical indications for HCT expanded and transplantation procedures improved, leading to an increased number of long-term survivors [2, 3]. Still, HCT is associated with considerable long-term morbidity, and development of oral complications is frequently reported [4, 5].

Some of these oral complications might be related to changes in salivary secretion. Unstimulated whole saliva (UWS), present in the oral cavity in resting conditions, is a viscous fluid mainly produced by the submandibular glands. The more watery stimulated whole saliva (SWS) is mainly produced by the parotid glands; the production of SWS is initiated by stimuli like chewing, taste and smell [6]. Both types of saliva are essential in maintaining oral health by protecting teeth and oropharyngeal mucosa and maintaining a balanced microbiota [6, 7]. In longitudinal studies, a decline in UWS [8, 9] and in SWS flow rates was reported post-HCT [9–12]. SWS flow rates tend to increase again over time [11, 13], while long-term data on UWS flow rates are lacking. Furthermore, salivary flow rates in HCT recipients are lowered compared to healthy controls [11, 12, 14]. This decline imposes several risks to the oral cavity, potentially resulting in caries, periodontitis and tooth loss [15].

High-intensity conditioning regimens might result in dysfunction of the major salivary glands [16] and the secretion rate from the minor salivary glands might be reduced as a result of chemotherapy [17]. Nevertheless, an association between the intensity of the conditioning regimen and reduction in salivary flow rate could not be established so far. Patients treated with high intensity conditioning demonstrated a tendency towards increasing prevalence of hyposalivation [11], while the intensity of the conditioning regimen was not related to SWS flow rates in regression analyses [12]. TBI as part of the conditioning resulted in a delayed recovery of SWS flow rates post-HCT [11].

Polypharmacy is a well-known risk indicator in the development of hyposalivation [6], and might be an explanation for the decline in salivary flow rates in HCT recipients. It was reported that an average of four different medications was used concomitantly by HCT recipients [12], and that 91% of the allogeneic HCT recipients in that study used medications that were known to reduce salivary flow rate [18]. Nevertheless, the number of medications nor the examined pharmaceutical groups were significantly associated with decreased SWS flow rates [12].

Chronic graft-versus-host disease (cGvHD), a complication from allogeneic transplantations, is an immune response of donor-derived cells against recipient tissues [1]. cGvHD is associated with histopathological changes in salivary glands, a reduction in salivary flow rate, and changes in the composition of saliva [19]. Several studies showed a persistently low salivary flow rate in cGvHD patients with virtually no recovery [14, 20], in contrast to allogeneic patients who did not develop cGvHD and autologous HCT recipients. It was suggested that salivary involvement in cGvHD might be irreversible [21]. More recent studies concluded that the effect of cGvHD on SWS flow rate was negligible [12] and that oral mucosal cGvHD was not related to UWS flow rate [22].

We aim to describe the development of hyposalivation of both UWS and SWS over time in HCT recipients, assessing both the period early post-HCT and the long term up to 18 months post treatment. The effect of several risk indicators in the development of hyposalivation will be determined.

## Methods

This study is an ancillary study of the Orastem study, a multinational, prospective, observational, longitudinal study on the impact of oral side effects from conditioning therapy before HCT [23]. Adult patients (≥ 18 years old) scheduled to receive an autologous or allogeneic HCT at Amsterdam University Medical Center, location AMC or Radboud University Medical Center (Radboudumc) Nijmegen were included. Patients scheduled for allogeneic HCT were eligible for inclusion independent of their diagnosis, while those scheduled for autologous HCT were eligible if diagnosed with multiple myeloma. Patients were excluded if they were not able to understand the provided information, a second HCT was planned in advance or if the time before HCT was too short to consider study participation. This study was registered in the Netherlands trial register (NL5645), approval was obtained by the Medical Research Ethical Committee (NL52117.018.15), and the study was conducted according to GCP guidelines and the World Medical Association Declaration of Helsinki. Before participating, all patients signed informed consent.

### Saliva collection

Saliva was collected at the baseline screening preceding the conditioning regimen, and once a week during the first 28 days following HCT while most patients were hospitalised. This resulted in a median of 2 samples (range: 1–4) per patient early post-HCT. All patients underwent saliva collections after 3 and 12 months, and allogeneic HCT recipients had additional saliva collections 6 and 18 months post-HCT.

The protocols for the collection of whole saliva were based on the guidelines for saliva collection of the University of Southern California School of Dentistry [24]. Patients were asked to refrain from eating, drinking, toothbrushing, and use of chewing gum 1 h before the collection. The collection of UWS started immediately after one swallow. Patients were asked to spit the saliva in a pre-weighed plastic cup for 5 min without making any effort to increase the salivary flow. During the collection of SWS, patients chewed on a piece of neutral chewing gum base. SWS was collected for 2–5 min, and the collection was preceded by swallowing after 1 min of chewing. Directly after collection, samples were weighted and flow rates were estimated by assuming 1 g of saliva equals 1 mL. Hyposalivation of UWS was defined as a flow rate of < 0.2 mL/min, and hyposalivation of SWS as < 0.7 mL/min [25]. Patients that had a flow rate below this threshold at least once during the first 28 days post-HCT, were classified as having hyposalivation early post-HCT.

### Medication data

Prescribed medications were extracted from the electronic medical record system (EPIC) in both centres. All prescribed medications that the patients were on, in the week preceding the pre-conditioning screening, were extracted. Only when medications during the pre-conditioning screening were not available in EPIC, patient-reported medications were used. Besides, medications were extracted from medical records for all patients that were hospitalised in the Amsterdam UMC, location AMC or the Radboudumc at least 7 days following HCT. Data on prescribed medications were available until resolution of neutropenia or discharge from the hospital. All different systemically administered medications (oral, intravenous, subcutaneous, sublingual, transdermal, rectal, inhalation) were counted; doses were not taken into account. Medications were divided into the following categories:

- Antimicrobials: antibiotics, antifungals, antiviral medications
- Supportive medication: sleep medication, antidepressants, anxiolytics, antacids, antiemetics, analgesics, antihistamines, laxatives, diuretics
- Anticancer and immunosuppressive medication: cytostatics, oncolytics, colony stimulating factors, corticosteroids, other immunosuppressives, protein kinase inhibitors
- Other medication

### Oral mucosal cGvHD

Oral mucosal changes related to cGvHD in allogeneic HCT recipients were determined by experienced dentists according to National Institutes of Health (NIH) Oral Mucosal Scale [26]. The severity of the three most common manifestations of oral cGvHD was evaluated. Erythema (scored 0–3), lichenoid lesions (scored 0–3), and ulcers (scored 0–6) were added up, resulting in a score between 0 and 12 [27]. Patients with an NIH OMS ≥ 2 were assigned as having oral mucosal cGvHD. Oral mucosal changes were evaluated after 3, 6, 12. and 18 months.

### Data analysis

The development of hyposalivation of UWS and SWS over time is graphically shown. UWS and SWS flow rates are shown from a subgroup of the present population: only patients from the Radboudumc are included due to higher precision salivary measurements performed in this centre. Salivary flow rates measured during the 4 weeks following HCT were combined, resulting in a mean score early post-HCT. Paired *t*-tests were used to determine changes in flow rates with measurements pre-conditioning. Statistical analyses were performed in R (version 4.1.3) and SPSS (version 27). Line and bar charts were made using GraphPad Prism (version 9.5.0).

### Risk indicators

Separate logistic regression models were built to study the influence of the following risk indicators on hyposalivation of UWS and SWS:

1. intensity of the conditioning regimen: a distinction was made between high intensity or myeloablative (MAC) conditioning regimens, and non-myeloablative or reduced intensity or (NMA/RIC) conditioning regimens [28]
2. TBI (yes vs no) as part of the conditioning
3. type of HCT (allogeneic vs autologous)
4. oral mucosal changes related to cGvHD after 3, 6, 12, and 18 months
5. number of prescribed medications during hospitalisation

The influence of the conditioning regimen and type of transplantation (analysis 1, 2 and 3) was determined early post-HCT and after 3 and 12 months. Analysis number four aimed to study the effect of oral mucosal changes related to cGvHD and simultaneous diagnosis of hyposalivation. In this analysis, one measurement out of four (3, 6, 12, or 18 months) was selected per patient. The first moment a patient developed oral mucosal cGvHD or hyposalivation was selected; the last measurement was used when patients did not develop hyposalivation or oral mucosal cGvHD. The fifth analysis focused on the effect of the number of prescribed medications during the hospitalisation phase, and contemporary hyposalivation. Patients who left the Amsterdam UMC, location AMC or Radboudumc within 7 days after the transplantation, because of transfer to a general hospital or discharge, were not included in this analysis.

Crude models included the above-mentioned risk indicators as only independent variable, while potential confounding factors were added to the adjusted models. The following covariates were considered for inclusion: age, sex, centre of treatment, comorbidities (yes vs no), and pre-conditioning hyposalivation (yes vs no). In fifth analysis, aiming to determine the effect of the number of medications on hyposalivation during hospitalisation, the length of the hospital stay was added as a covariate. The number of covariates in the analysis was restricted based on the extent to which the odds ratio (OR) was affected, resulting in the exclusion of variables with a negligible effect. Results of the analysis are graphically shown as ORs with 95% confidence intervals (95% CI).

## Results

**Fig. 1:**
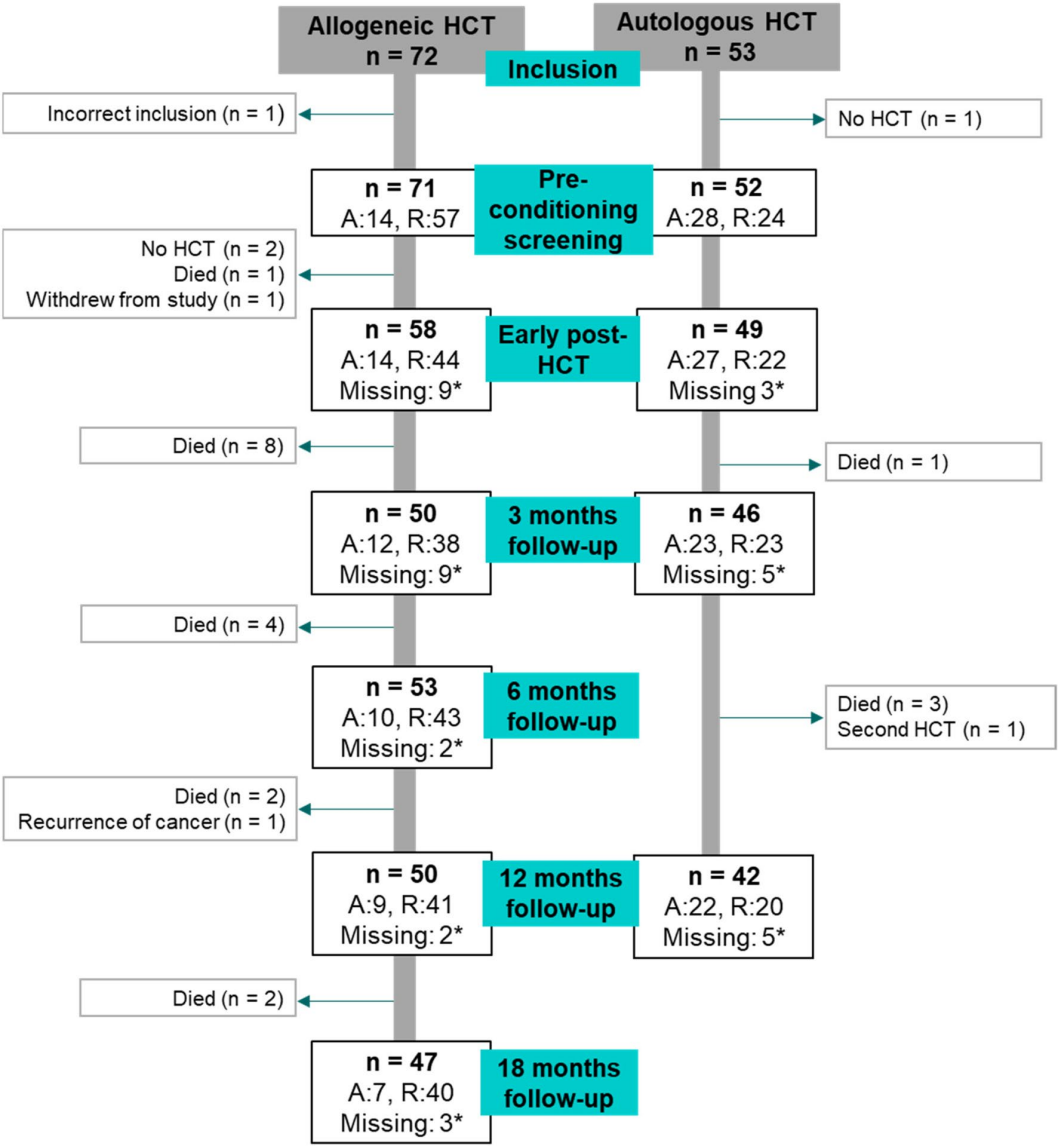
Flowchart of the study. A distinction is made between patients treated at Amsterdam UMC, location AMC (A) and Radboudumc (R). Reasons for exclusion and irreversible loss to follow-up are shown in the grey squares on the left and right side of this diagram. In 12 patients, no saliva was collected early post-HCT because patients were ill/nauseous (*n* = 5), the hospital stay was too short (*n* = 6), or unknown reasons (*n* = 1). Reasons for 26 incidental missed appointments in 23 patients during the long-term follow-up, marked with asterisk in this diagram were the following: unable to come due to hospitalisation, rehabilitation, or illness (*n* = 4), refused to come or did not come (*n* = 10), unreachable (*n* = 3), or other/unknown reasons (*n* = 9)

In total, 125 patients that were planned for HCT signed informed consent and were included between September 2015 and October 2017. At least one salivary measurement was performed in 107 patients early post-HCT and of these, 86 patients were hospitalised in the Amsterdam UMC, location AMC or Radboudumc for more than 7 days post-HCT. During the study period, 21 HCT recipients (17%) died. The number of patients present at different follow-ups and reasons for loss to follow-up are shown in Fig. 1. The median age of autologous and allogeneic NMA/RIC recipients was 59 years, while allogeneic MAC recipients were younger (median 44.5 years). Baseline characteristics and HCT-related characteristics of the participants are reported in Table 1.

**Table 1:** Baseline characteristics of HCT recipients

	Autologous (52)	Allogeneic MAC* (14)	Allogeneic NMA/RIC* (53)
Median age in years (range)	59 (33–69)	44.5 (23–55)	59 (19–74)
Gender, *n* (%) female	24 (46%)	7 (50%)	23 (43%)
Centre			
Amsterdam UMC, location AMC, *n*	28	3	11
Radboudumc, *n*	24	11	42
Diagnoses, n			
Acute myeloid leukaemia		8	20
Acute lymphoblastic leukaemia		4	1
Lymphoma		1	7
Chronic lymphocytic leukaemia			3
Myelodysplastic syndrome			9
Chronic myeloid leukaemia		1	2
Myelofibrosis			4
Severe aplastic anaemia			2
Multiple myeloma	52		2
Other			3
Comorbidities (≥ 1 current medical conditions, other than the diagnoses above), n (%)	25 (48%)	2 (14%)	15 (28%)
Earlier radiation therapy to head and neck region, *n* (%)	0	0	3 (6%)
Median (range) number of prescribed medications during the week preceding the pre-conditioning screening	6 (0–17)	2 (0–9)	4 (0–15)
Conditioning:			
Myeloablative, *n*	52	14	
Reduced intensity, *n*			24
Nonmyeloablative, *n*			29
Total body irradiation, *n*		13	32
No total body irradiation, *n*	52	1	21
Donor:			
Unrelated donor, match		8	36
Unrelated donor, mismatch			3
Sibling donor		6	10

^*^Four allogeneic HCT recipients who were excluded before the conditioning regimen are not included in this table

### Hyposalivation and salivary flow rate

**Fig. 2:**
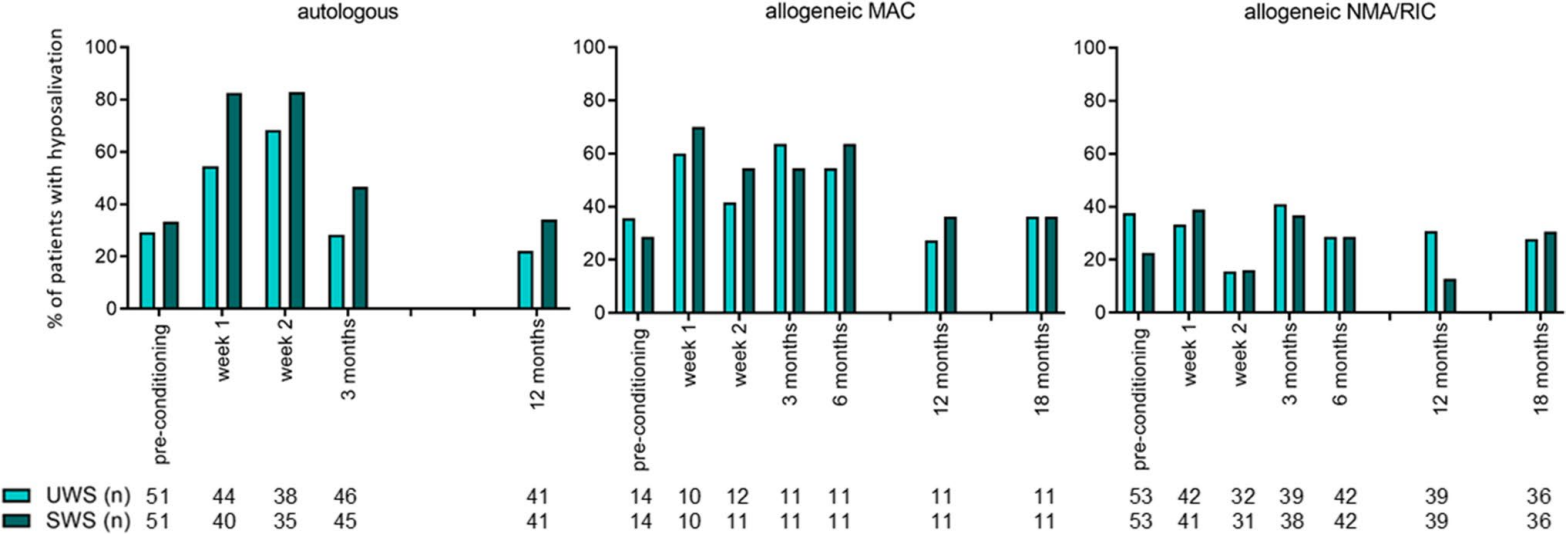
Prevalence of hyposalivation over time. Numbers (*n*) are the numbers of patients that contributed one or more saliva samples per time point or period. Five SWS samples in two patients are missing due to prosthesis, and 2 UWS samples in one patient are missing due to chewing gum use preceding the collection. Furthermore, several patients felt too ill or nauseous, or experienced too much pain in the oral cavity to collect SWS early post-HCT. Abbreviations: UWS, unstimulated whole saliva; SWS, stimulated whole saliva; MAC, myeloablative conditioning; NMA/RIC, non-myeloablative or reduced intensity conditioning

Saliva samples were collected between 8.30 a.m. and 16.30 p.m. Pre-conditioning, 34% of the patients was diagnosed with hyposalivation of UWS and 29% of SWS. This number increased to 54% and 67% early post-HCT, and diminished to 26% and 25% 12 months post-HCT respectively. The percentage of patients with hyposalivation of UWS and SWS is shown in Fig. 2. The increase in hyposalivation early post-HCT was most pronounced in the autologous HCT recipients; in allogeneic recipients receiving an NMA/RIC conditioning, only limited changes over time were seen.

**Fig. 3:**
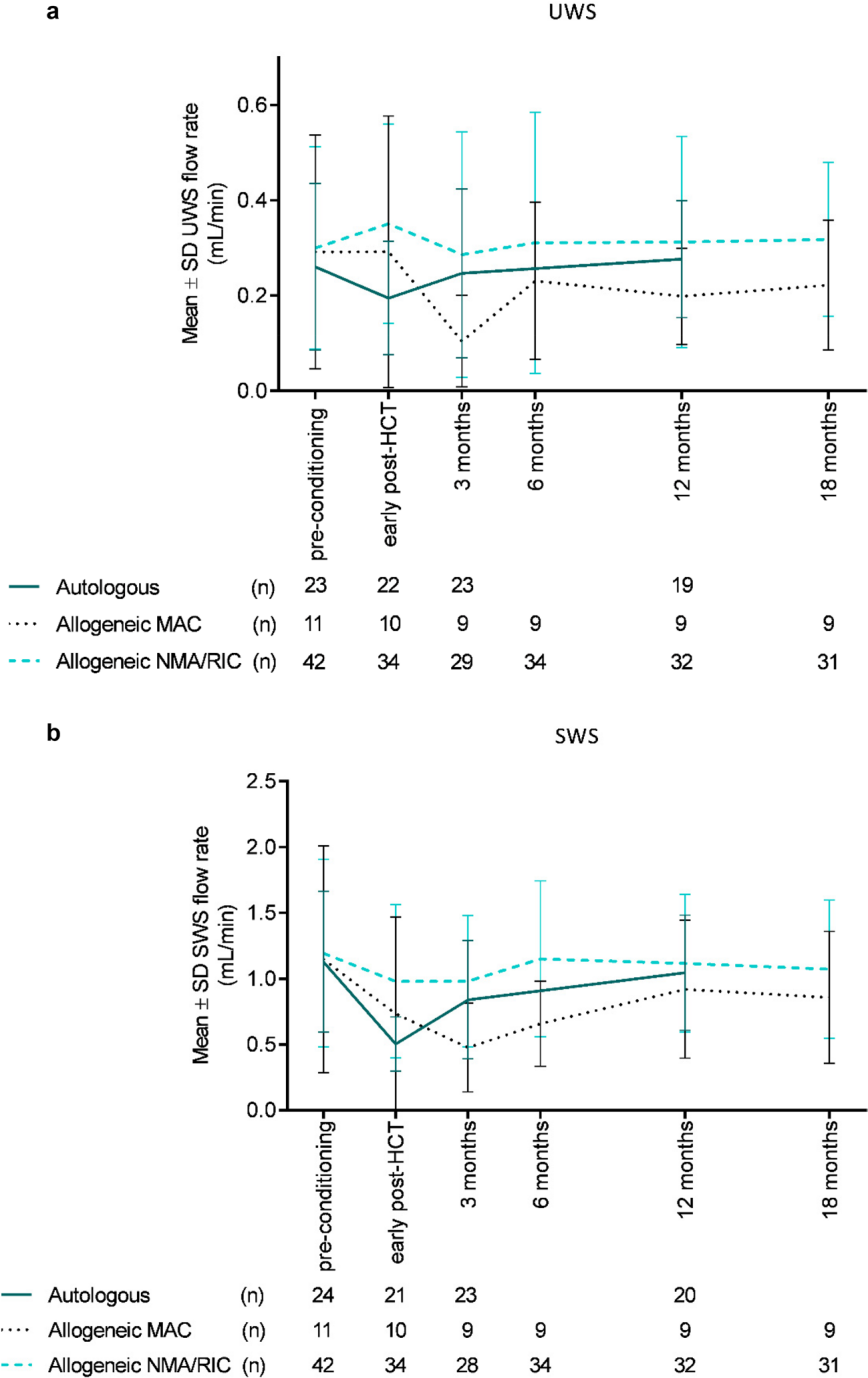
Mean salivary unstimulated whole saliva (UWS, **a**) and salivary stimulated whole saliva (SWS, **b**) flow rates with standard deviations (SD) over time. Numbers of patients (*n*) who contributed saliva sample(s) per time point or period are listed below the graphs. Abbreviations: MAC, myeloablative conditioning; NMA/RIC, non-myeloablative or reduced intensity conditioning

Salivary flow rates from patients treated at the Radboudumc are shown in Fig. 3a (UWS) and b (SWS). Looking at all patients, SWS flow rates declined the first week after treatment with 0.44 mL/min (95% CI: 0.29–0.58). Flow rates were still reduced 3 months post-HCT (mean decline from baseline: 0.26 mL/min, 95% CI: 0.11–0.41). Twelve months post-HCT, the difference was not statistically significant anymore (mean: 0.11, 95% CI: − 0.03–0.26). The reduction shortly after treatment was most pronounced in the two groups receiving a myeloablative conditioning regimen (autologous and allogeneic MAC). In the autologous subgroup, flow rates started to increase again after the first month. This increase was seen after 3 months for the allogeneic groups.

UWS flow rates seem to follow the same trend over time as SWS, but changes from baseline were less pronounced and did not reach statistical significance. In the allogeneic subgroup receiving NMA/RIC, only limited changes in mean scores were seen. The drop in flow rate for autologous patients was most pronounced early post-HCT, and for allogeneic patients receiving MAC it was most pronounced after 3 months.

### Prescribed medications

**Fig. 4:**
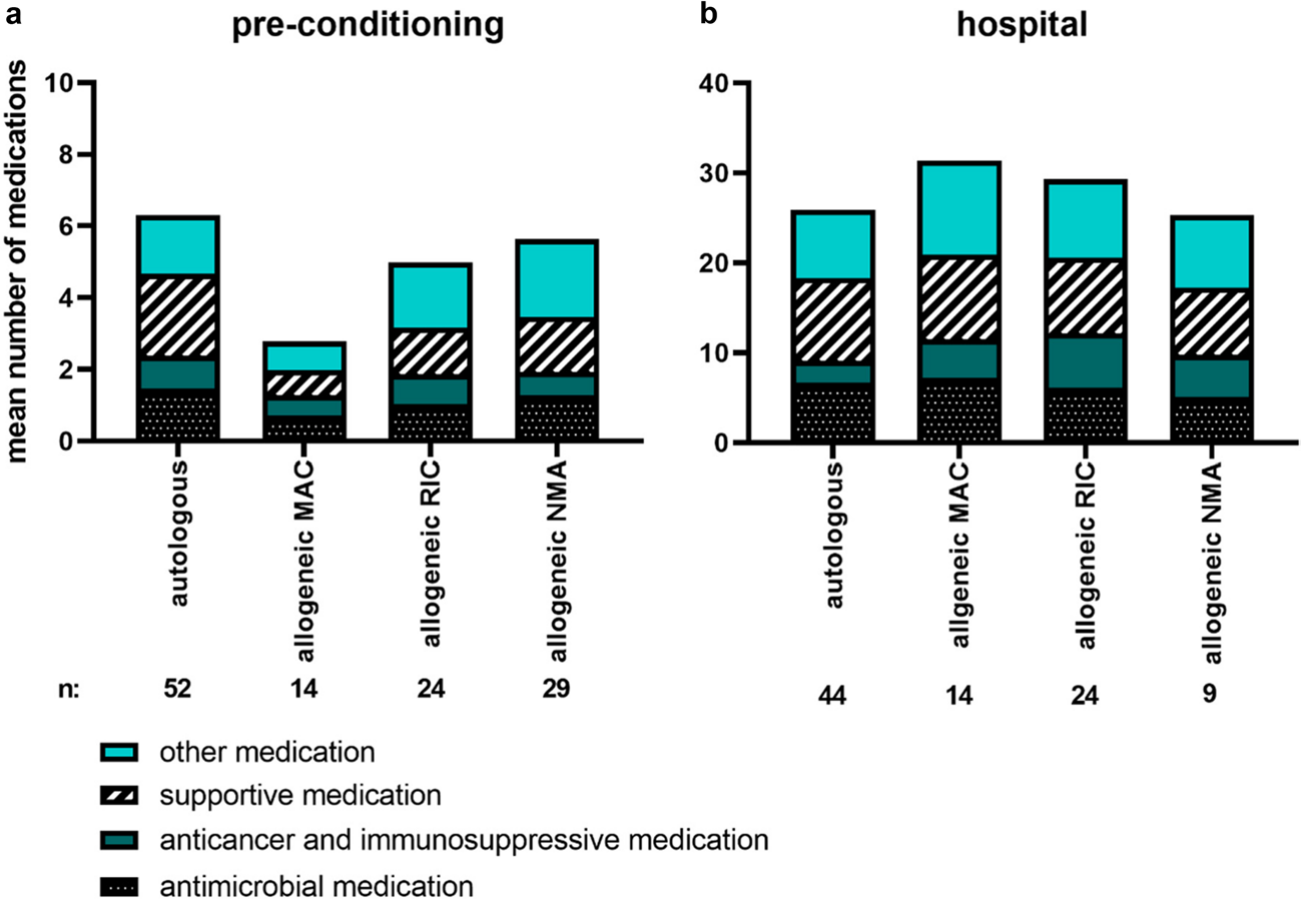
Mean number of prescribed medications during the week preceding the pre-conditioning screening (**a**), and during the hospitalisation phase (**b**). Numbers of patients (*n*) are listed below the graph. Autologous HCT recipients stayed for median 19 days (range: 15–32) in the hospital, allogeneic myeloablative (MAC) recipients for 24 days (range: 17–33), reduced intensity (RIC) for 24.5 days (range 20–35), and non-myeloablative (NMA) for 16 days (range 14–23)

During the week preceding the pre-conditioning screening, 9 patients (7%) did not use any medication, while 60 patients (49%) used ≥ 5 medications. In total, 59 patients (52%) used anticancer or immunosuppressive medication in this week. Mean numbers of prescribed medications for each category and subgroup in the week preceding the pre-conditioning screening can be found in Fig. 4a.

In total, 27 patients were discharged in the week following HCT. From the remaining patients, medication data was available for a median of 21 days (range: 14–35 days). During this hospital stay, patients used a median of 27 (range: 16–45) different medications. This number of medications includes the conditioning regimen that was administered at the beginning of hospitalisation. Patients used on average 6 different antimicrobial medications (range: 3–15) and 8 different supportive medications (range: 4–16). Mean numbers of prescribed medications for each category and subgroup during hospitalisation are shown in Fig. 4b.

### Oral mucosal cGvHD

**Fig. 5:**
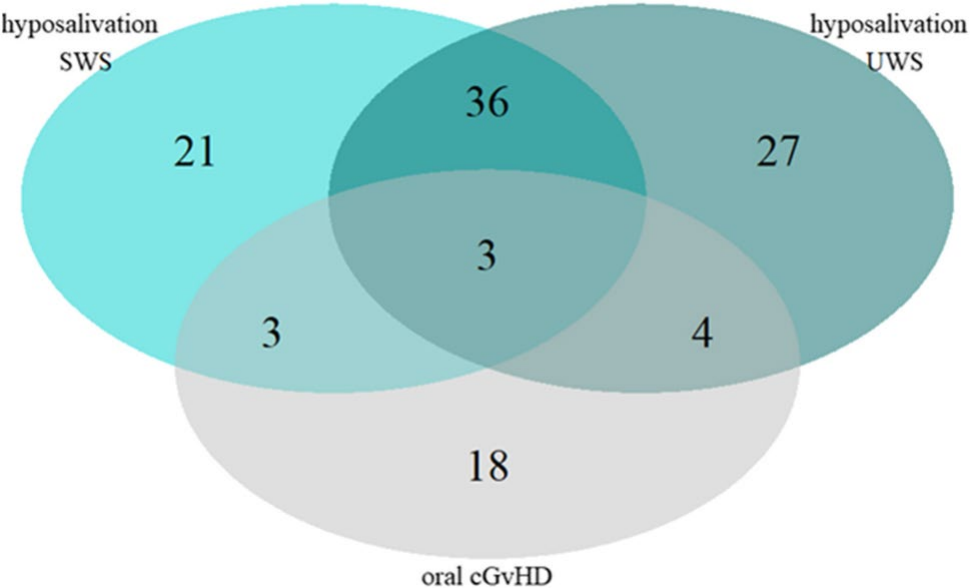
Venn diagram showing the diagnoses of hyposalivation of stimulated whole saliva (SWS), unstimulated whole saliva (UWS), and oral mucosal chronic graft-versus-host disease (cGvHD)

Oral mucosal changes related to cGvHD were seen 28 times in 15 patients (25%). At these 28 occasions, the median NIH OMS was 3 (range 2–8). At the 3 months follow-up visit, oral cGvHD-related mucosal changes were seen in 3 patients, after 6 months in 12 patients, after 12 months in 7 patients, and after 18 months in 6 patients. The majority of patients had no hyposalivation at the visit when mucosal changes were seen (Fig. 5).

Data of all allogeneic patients seen after 3, 6, 12, and 18 months are combined. Overlap indicates simultaneous diagnoses.

### Risk indicators

**Fig. 6:**
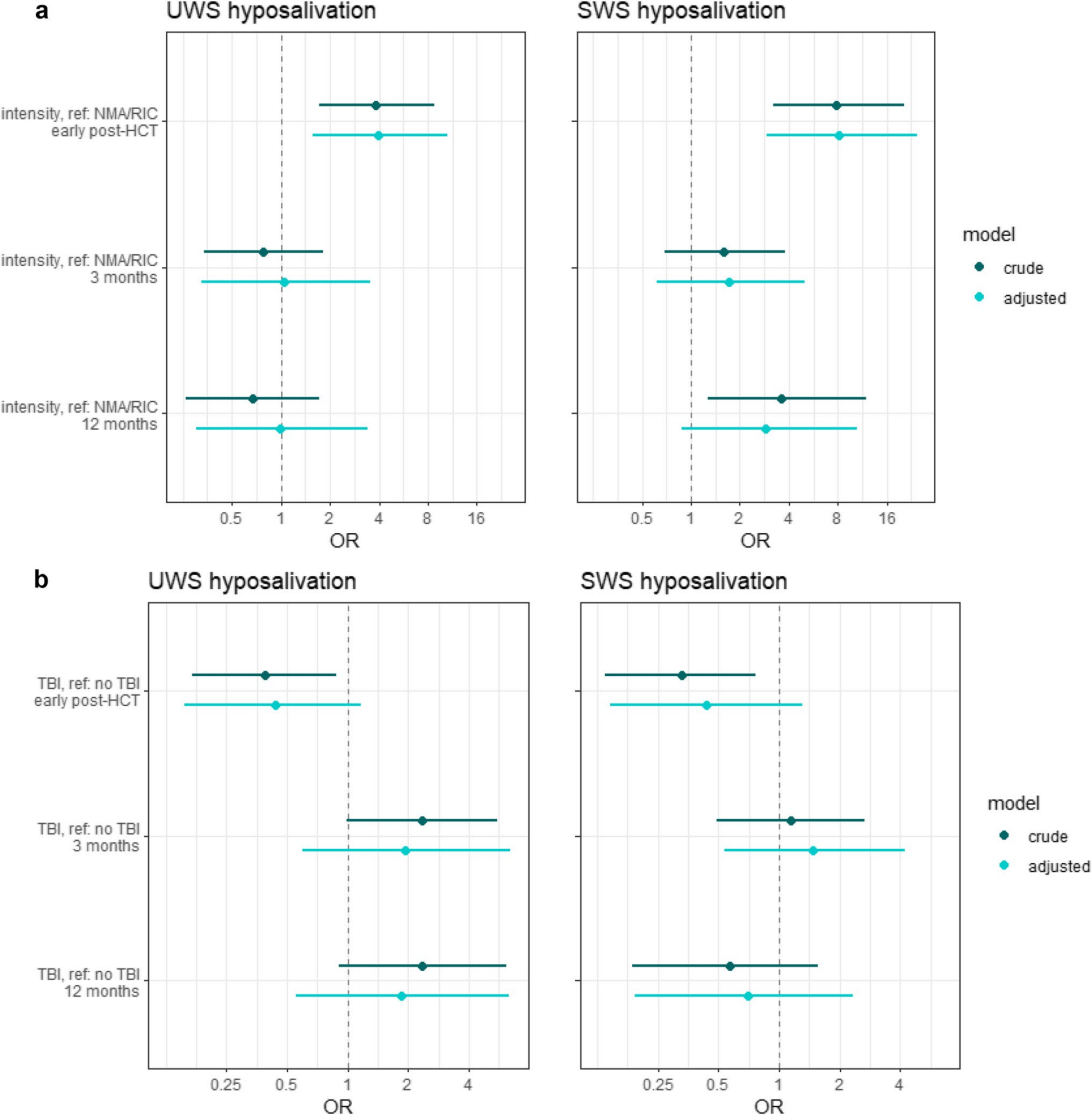

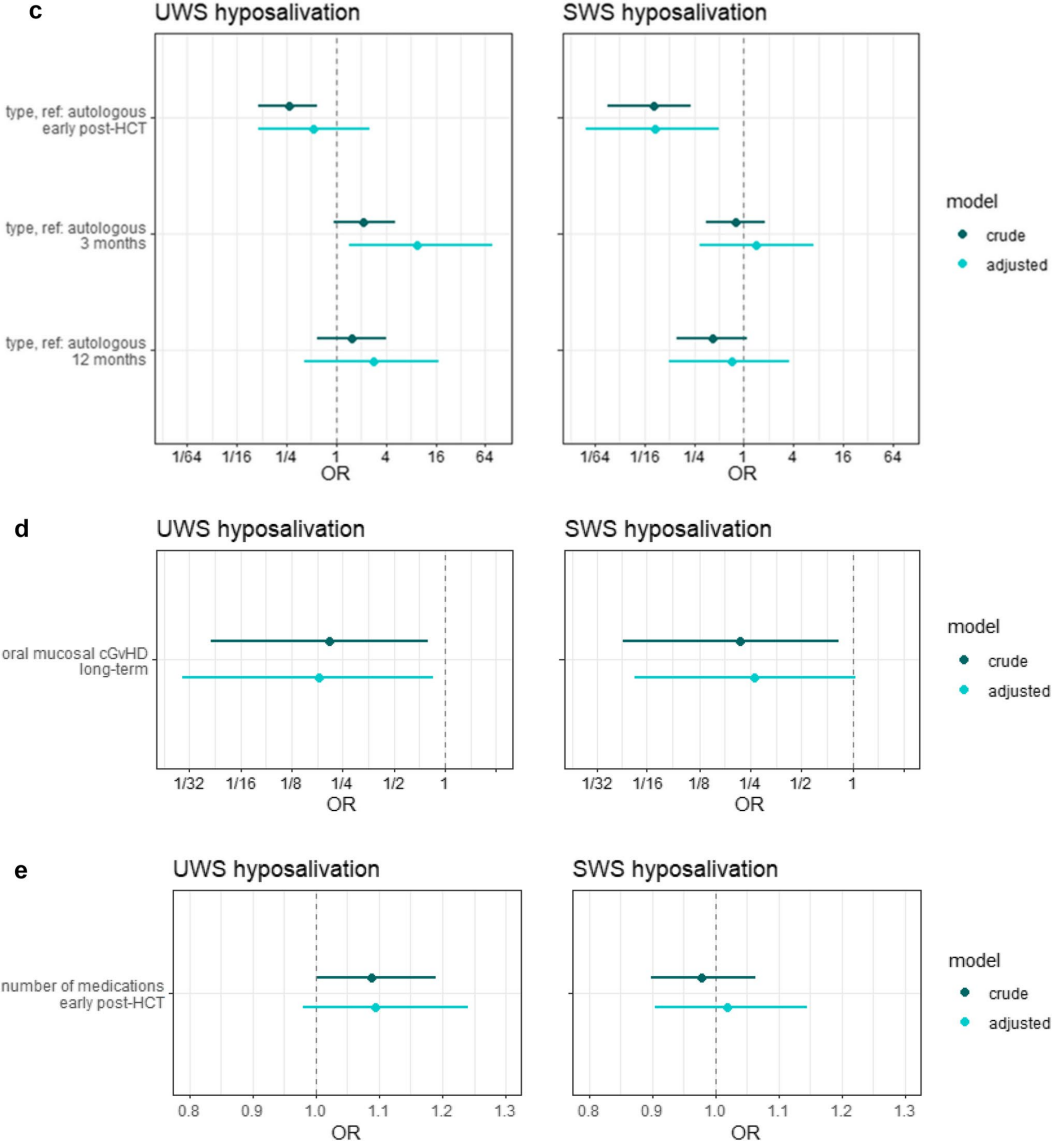
The relation between several risk indicators and hyposalivation. Hyposalivation of unstimulated whole saliva (UWS) is shown on the left side, hyposalivation of stimulated whole saliva (SWS) on the right side. Odds ratios (OR) are shown with their 95% confidence interfalls. **a** Relation between intensity of the conditioning regimen and hyposalivation at different moments in time Myeloablative conditioning regimens are compared to non-myeloablative or reduced intensity (NMA/RIC) conditioning regimens. In the adjusted model, the following variables were added: hyposalivation at baseline (UWS and SWS respectively), total body irradiation, and age.** b** Relation between total body irradiation (TBI) as part of the conditioning regimen and hyposalivation at different moments in time. In the adjusted model, the following variables were added: hyposalivation at baseline (UWS and SWS respectively), the intensity of the conditioning regimen and age. **c** The relation between the type of transplantation (allogeneic vs autologous) and hyposalivation at different moments in time. In the adjusted model, the following variables were added: the intensity of the conditioning regimen, hyposalivation at baseline (UWS and SWS respectively) and age. **d** Relation between hyposalivation and oral mucosal changes related to chronic graft-versus-host disease (cGvHD) at the same follow-up. In the adjusted model, the following variables were added: hyposalivation at baseline (UWS and SWS respectively), the intensity of the conditioning regimen and age. **e** The relation between the number of prescribed medications during hospitalisation and hyposalivation early post-HCT. In the adjusted model, the following variables were added: the intensity of the conditioning regimen, hyposalivation at baseline (UWS and SWS respectively), length of hospital stay in days and age

The association between the intensity of the conditioning regimen and hyposalivation is shown in Fig. 6a. Early post-HCT, the intensity was a significant risk indicator in the development of hyposalivation of both UWS and SWS. MAC recipients had, after adjusting, a 3.9 (95% CI: 1.6–10.6) times higher odds of developing hyposalivation of UWS, and an 8.2 (95% CI: 2.9–24.6) times higher odds developing hyposalivation of SWS. After 3 and 12 months, the influence of the intensity of the conditioning regimen on hyposalivation of SWS diminished to non-significant levels. An effect of the intensity on hyposalivation of UWS after 3 and 12 months was lacking.

The association between TBI as part of the conditioning regimen and hyposalivation is shown in Fig. 6b. After adjusting for confounding factors, TBI was not significantly related to hyposalivation of UWS or SWS at any moment. TBI receivers tended to have more hyposalivation of UWS after 3 and 12 months, but this difference did not reach statistical significance.

The association between the type of transplantation and hyposalivation is shown in Fig. 6c. Autologous HCT recipients had more hyposalivation of SWS early post-HCT (OR: 0.08; 95% CI: 0.01–0.5), while allogeneic HCT recipients had more hyposalivation of UWS 3 months post treatment (OR: 9.6; 95% CI: 1.4–77). After 12 months, no significant effect remained.

The association between oral mucosal cGvHD and hyposalivation is shown in Fig. 6d. ORs of below 1, both in the crude and the adjusted models, confirm that oral mucosal changes and hyposalivation do not occur more often simultaneously.

The association between the number of prescribed medications during hospitalisation and hyposalivation early post-HCT is shown in Fig. 6e. No association was found between the number of medications and hyposalivation of SWS. The odds of developing hyposalivation of UWS was 1.1 (95% CI: 1.0–1.2) times higher for every additional medication that was prescribed.

## Discussion

The aim of this prospective longitudinal study was to describe the development of hyposalivation over time in HCT recipients, and determine risk indicators. Hyposalivation affected the majority of patients early post-HCT. The intensity of the conditioning regimen was a significant risk indicator in the early post-HCT development of hyposalivation. Autologous HCT recipients had more hyposalivation of SWS early post-HCT, while allogeneic HCT recipients had more hyposalivation of UWS 3 months post treatment. Nor TBI as part of the conditioning regimen, the number of prescribed medications or mucosal oral cGvHD worsened hyposalivation significantly.

The intensity of the conditioning regimen was a significant risk indicator in the development of hyposalivation of both UWS and SWS early post-HCT. This effect was not significant anymore after 3 and 12 months. It was suggested that chemotherapy impaired both acinar and ductal function of salivary gland tissue [29]. The finding that MAC-recipients had more hyposalivation than RIC-recipients, confirms the causal relation between the conditioning regimen and salivary dysfunction. Previous studies concluded that patients treated with MAC demonstrated a non-significant tendency for an increasing prevalence of hyposalivation 6 months post-HCT [11], or found no relation between the intensity of the conditioning regimen and hyposalivation [12, 22]. None of these studies measured hyposalivation early post-HCT, and might therefore have underestimated the association between intensity and hyposalivation.

No significant relation between TBI and hyposalivation could be established in the current study. Subjects in the current study received a dose between 2 and 9 Gray, which might not have reached the threshold above which salivary gland function will diminish [30]. It was reported before that recovery of salivary flow rate was slower after administration of TBI [11]. Other studies found no association between salivary hypofunction and TBI [5, 22].

Autologous HCT recipients had a 12 times higher odds (OR: 0.08) of developing hyposalivation of SWS early post-HCT, compared to allogeneic recipients, even after adjusting for confounding factors like the intensity of the conditioning regimen. The autologous HCT recipients included in the current study comprise a homogeneous population: all patients were diagnosed with multiple myeloma and received high dose melphalan as conditioning regimen. Melphalan is one of the chemotherapeutic drugs that is actively secreted by the salivary glands [31], and might therefore be related to an increased reduction in saliva secretion early post-HCT.

In the long term, allogeneic recipients had more hyposalivation of UWS than autologous recipients, a difference that reached significance after 3 months. This increased prevalence of hyposalivation might be explained by histopathological changes in the salivary glands caused by cGvHD [19]. Nevertheless, the majority of patients with long-term hyposalivation had no oral mucosal cGvHD simultaneously. This finding is in agreement with literature, and supports the suggestion that salivary gland involvement and oral mucosal cGvHD are common and clinically distinct manifestations of cGvHD [22, 32]. Patients with oral mucosal cGvHD even tended to have less hyposalivation. We hypothesize that pain caused by mucosal changes could be related to an increased salivary flow rate, as is seen in other potentially pain inducing conditions or situations of the oral mucosa, like teething [33], and eating spicy foods [34].

HCT recipients used a median of 27 (range: 16–45) different medications during hospitalisation. Because polypharmacy is a well-known risk indicator in the development of hyposalivation [6], it is not surprising that the majority of patients developed hyposalivation early post-HCT. The number of medications had a non-significant effect on hyposalivation of UWS, and the number of medications was not related to hyposalivation of SWS. It is reported in literature that in medication-induced salivary gland hypofunction, UWS flow rate was usually reduced, whereas SWS flow rates were within the normal range [6]. A potential effect of the medication might be neglected in the current analysis, because the dose and type of the prescribed medications were not taken into account. Previous publications could also not find an association between the number of medications and decreased SWS [12] or UWS [18] flow rates post-HCT.

A limitation of the current study is the extensive variation in the time of day at which saliva was collected, resulting in a lower precision due to the circadian rhythm [35]. Furthermore, the limited number of patients made statistical analysis within the subgroups (e.g. autologous or allogeneic) not meaningful. Nevertheless, clear trends in salivary flow rates over time are seen, that are in agreement with literature. The previous reported lowered UWS flow rates 3 months [8] and lowered SWS flow rates 6 months post allogeneic HCT [11, 13], and recovery after 12 months [11, 13], is in agreement with our results. SWS flow rates decreased shortly after HCT while only limited changes in UWS flow rates were seen at the same time, which is also in agreement with literature [9, 10, 36]. One previous publication reported even an increased UWS flow rate in allogeneic HCT recipients early post-HCT [8].

More than half of the HCT recipients was diagnosed with hyposalivation early post-HCT; a quarter still had hyposalivation after 12 months. These numbers are high compared to the prevalence of hyposalivation in the general population, that was estimated to be 20% (95% CI: 15–25) [37]. According to literature, the average UWS flow rate ranges between 0.3 and 0.4 mL/min, and the mean SWS flow rate between 1.5 and 2 mL/min [6]. Compared to these values, salivary flow rates in HCT recipients were already low pre-conditioning, and remained lowered after recovery in the long term. Previous publications confirmed that SWS flow rates in HCT recipients remained lowered 6 and 12 months post treatment compared to healthy controls [11, 12].

A sufficient amount of saliva is essential to maintain oral health, and therefore, HCT recipients will have an increased risk for dental caries [38] and complaints of mouth dryness. We recommend treating dentists to be aware of the high prevalence of hyposalivation in HCT recipients, and the increased risk of oral complications. It may be reasonable to plan additional oral check-ups and consider additional preventive strategies.

## Data Availability

The data that support the findings of this study are available under request from the first author.
